# The effect of gut microbiome-targeted therapies in nonalcoholic fatty liver disease: a systematic review and network meta-analysis

**DOI:** 10.3389/fnut.2024.1470185

**Published:** 2025-01-06

**Authors:** Yijia Song, Sutong Liu, Lihui Zhang, Wenxia Zhao, Yuanmei Qin, Minghao Liu

**Affiliations:** ^1^Department of Spleen, Stomach, Hepatobiliary Disease, The First Affiliated Hospital of Henan University of Chinese Medicine, Zhengzhou, China; ^2^The First Clinical Medical School of Henan University of Chinese Medicine, Zhengzhou, China; ^3^The Nursing School of Henan University of Chinese Medicine, Zhengzhou, China

**Keywords:** non-alcoholic fatty liver disease, microbiome-targeted therapies, network meta-analysis, fecal microbiota transplant, antibiotics, probiotics, synbiotics, prebiotics

## Abstract

**Background:**

The incidence of NAFLD is increasing. Preclinical evidences indicate that modulation of the gut microbiome could be a promising target in nonalcoholic fatty liver disease.

**Method:**

A systematic review and network meta-analysis was conducted to compare the effect of probiotics, synbiotics, prebiotics, fecal microbiota transplant, and antibiotics on the liver-enzyme, metabolic effects and liver-specific in patients with NAFLD. The randomized controlled trails (RCTs), limited to English language were searched from database such as Pubmed, Embase, Web of science and Cochrane Library from inception to November 2024. Review Manager 5.3 was used to to draw a Cochrane bias risk. Inconsistency test and publication-bias were assessed by Stata 14.0. Random effect model was used to assemble direct and indirect evidences. The effects of the intervention were presented as mean differences with 95% confidence interval.

**Results:**

A total of 1921 patients from 37 RCTs were eventually included in our study. 23 RCTs evaluated probiotics, 10 RCTs evaluated synbiotics, 4 RCTs evaluated prebiotics, 3 RCTs evaluated FMT and one RCT evaluated antibiotics. Probiotics and synbiotics were associated with a significantly reduction in alanine aminotransferase [ALT, (MD: −5.09; 95%CI: −9.79, −0.39), (MD: −7.38, 95CI%: −11.94, −2.82)] and liver stiffness measurement by elastograph [LSM, (MD: −0.37;95%CI: −0.49, −0.25), (MD: −1.00;95%CI: −1.59, −0.41)]. In addition to, synbiotics was superior to probiotics in reducing LSM. Synbiotics was associated with a significant reduction of Controlled Attenuation Parameter [CAP, (MD: −39.34; 95%CI: −74.73, −3.95)]. Both probiotics and synbiotics were associated with a significant reduction of aspartate transaminase [AST, (MD: −7.81; 95%CI: −15.49, −0.12), (MD: −13.32; 95%CI: −23, −3.64)]. Probiotics and Allogenic FMT was associated with a significant reduction of Homeostatic Model Assessment for Insulin Resistance [HOMA-IR, (MD: −0.7, 95%CI: −1.26, −0.15), (MD: −1.8, 95%CI: −3.53, − 0.07)]. Probiotics was associated with a significant reduction of body mass index [BMI, MD: −1.84, 95%CI: −3.35, −0.33].

**Conclusion:**

The supplement of synbiotics and probiotics maybe a promising way to improve liver-enzyme, LSM, and steatosis in patients with NAFLD. More randomized controlled trials are needed to determine the efficacy of FMT and antibiotics on NAFLD. And the incidence of adverse events of MTTs should be further explored.

**Systematic review registration:**

https://www.crd.york.ac.uk/prospero/, CRD42023450093.

## 1 Introduction

Nonalcoholic fatty liver disease (NAFLD) is a spectrum of liver tissue abnormalities that includes isolated hepatic steatosis, nonalcoholic steatohepatitis (NASH), liver fibrosis, cirrhosis, and liver cancer. With rapid economic development and lifestyle changes, NAFLD has become the leading cause of chronic liver disease, affecting 20–25% of adults worldwide, and estimated to affect 20% individuals with NASH (1). As a severe subtype of NAFLD, the incidence of NASH is increasing. It is projected to affect over 50% of the population by 2031, increasing incidence of liver cirrhosis, hepatocellular carcinoma, and mortality, imposing an economic burden on society (2, 3). There is no approved drug for NAFLD (4). Lifestyle modification, such as physical activity and weight loss, is a major intervention, that is difficult for overweight patients (5).

The gut contains a complex colony of trillions of microorganisms that live in harmony with the human body and help regulate metabolism, immunity, digestion, and nutrient absorption (6). Changes in the composition or function of the intestinal flora can participate in occurrence and development of various diseases by dysregulating host metabolism and immunity (7, 8). Preclinical studies have shown that regulating the gut microbiota can inhibit the development of obesity and hepatic steatosis, reduce liver inflammation, and delay the occurrence of NASH (9–11), indicating that targeting gut microbiota is a promising therapeutic measure.

Microbiome-targeted therapies (MTTs) have been proposed as a promising approach to regulate the gut microbiome, including several categories of antibiotics, probiotics, synbiotics, prebiotics and fecal microbiota transplantation (FMT). FMT is a process in which fecal flora collected from a healthy donor is transferred into a patient through a series of delivery routes, such as colonoscopy, nasogastric tube, and enema. Evidence shows that FMT could significantly reduce intracellular hepatic lipid and proinflammatory cytokine concentrations in high-fat diet-fed NAFLD mice and liver fibrosis and inflammatory infiltrates in NASH mice (12). In addition, Anecdotal. et al. also found a significant improvement in insulin sensitivity after the FMT from lean, healthy donors (13). Probiotics is live, non-pathogenic microorganisms that can improve gut health, as well as their critical metabolites. Acetate from probiotics could prevent NAFLD-HCC progression by binding with G-coupled protein receptor 43 (GPR43) and suppressing the IL-6/JAK1/STAT3 signaling pathway (14). Prebiotics can alleviate endotoxemia and inflammation, providing an alternative way to improve metabolic disorders. Beisner et al. found that prebiotics inulin and its fermentation product butyrate could attenuate weight gain and hepatic steatosis by promoting mucosal barrier integrity and strengthening paneth cell antimicrobial function (15). Synbiotics is a combination of probiotics and prebiotics. Alves et al. found that synbiotics supplementation could upregulate the expression of peroxisome proliferator-activated receptor *α* (PPAR-α) and downregulate sterol regulatory element-binding protein 1c (SREBP-1C) to reduce steatosis by increasing the *β*-oxidation process and modulating lipogenesis (16).

Recently, with increasing evidence from clinical trials demonstrating that MTTs play a significant role in improving NAFLD/NASH, the systematic review and meta-analysis by Sharpton et al. (17) was expanded to clearify the impact of probiotics, synbiotics, and prebiotics on NAFLD. However, due to the limited number of included literatures, only those on probiotics, synbiotics, and prebiotics were analyzed, and the efficacy and safety of MTTs in NAFLD treatment, including FMT and antibiotics, remains unevaluated. Moreover, few studies directly compare MTTs efficacy in NAFLD treatment (18). Therefore, given current literature limitations, our aim is to conduct a comprehensive systematic review and network meta-analysis, including as many pertinent literatures as possible, to evaluate the efficacy of MTTs in NAFLD/NASH treatment across hepatic inflammation, energy metabolism, and liver-specific outcomes.

## 2 Methods

This study follows the guidance of Preferred Reporting Items for meta-analysis and Systematic review of the network meta-analysis list. We established a protocol for the review, which was registered with PROSPERO prior to commencing the study^1^ (CRD42023450093).

## 3 Study selection

Included criteria: (1) study type: randomized controlled trails (RCTs); (2) Study object: NAFLD patients, which was defined by either Liver histology or noninvasive imagine modality (MRI, ultrasound, or elastography); (3) intervention measures: the experiment group was treated by MTTs, which was defined as interventions in any of the following 5 categories: prebiotics, synbiotics antibiotics, prebiotics and FMT. The control group was treat with placebo, usual care, and other MTTs different from the experiment group; (4) duration of therapy was≥4 week (excluding FMT trials); (5) Outcome indicators: one of the following outcomes was assessed: LSM, CAP, ALT, AST, TG, HDL-C, LDL-C, BMI, HOMA-IR. Excluded criteria: (1) hepatitis steatosis or fibrosis in patients were caused by hepatitis, liver cancer, autoimmune hepatitis, or other factors; (2) the study was not RCT; (3) the study did not acquire full text; and (4) the study was duplicated.

## 4 Search strategy

Pubmed Embase Web of science Cochrane Library were used as database for RCT research retrieval. The retrieval time was from the establishment of the database to November 2024. Keywords included were provided in Supplementary Table S1.

## 5 Data extraction and quality assessment

Two investigators independently read and screened the studies according to the inclusion criteria, and extracted data from the final included studies. The collected content included the authors of the included studies, the year of publication, the diagnostic criteria of the disease, the sample size, the age and gender of the participants, the intervention, follow-up duration, the outcomes, adverse reactions, and other relevant information. The mean and standard deviation (SD) values at the endpoint were directly extracted or calculated from the provided data. Cross-checks were conducted after screening, and a third party will be consulted to assist in judgment in case of disagreement.

The methodological quality of the included studies was assessed using the RCT risk of bias assessment tool in the Cochrane Handbook for Systematic Reviews. Two review authors independently evaluated each outcome in seven aspects: randomization sequence, allocation concealment, blinding of patients and staff, blinding of outcome assessors, completeness of outcome data, selective reporting of results, and other sources of bias. The risk of bias for each outcome was assessed as ‘low-risk’, ‘high-risk’, or ‘unclear’.

## 6 Statistical analysis

In this study, Review Manager 5.3 was used to draw a Cochrane bias risk. Stata 14.0 were used for network meta-analysis (NMA). We estimated summary mean difference (MD) for continuous outcomes using pairwise and network meta-analysis. The significance of an effect was expressed by 95% confidence interval (CI).

The results of included articles were described in the tables. Network evidence plots were used to show the relationship between interventions. In the Network evidence plots, the size of the dot represents the sample size of the treatment method. The larger dot is, the more the sample size is. The thickness of the line between two dots represents the number of studies. The thicker the line is, the more the number of studies is. The Surface Under the Cumulative Ranking (SUCRA) was used to reflect the probability order of different MTTs to be the best treatment option. A higher SUCRA score indicated a more effective or accepted treatment. Comparison adjusted funnel plots were used to assess the presence of publication bias. When there was a closed loop, we carried out inconsistency test. In the inconsistency test, if *p* < 0.05, it was considered that there existed inconsistency between the direction or indirection comparison. If there is an inconsistency, we chose random effect network meta-analysis model. We used the node splitting method to investigate the sources of inconsistency. Once node splitting method identifies the inconsistent node segments, we applied the stepwise exclusion method to pinpoint the specific studies or factors causing the inconsistency.

## 7 Results

### 7.1 Search results

**Figure 1:**
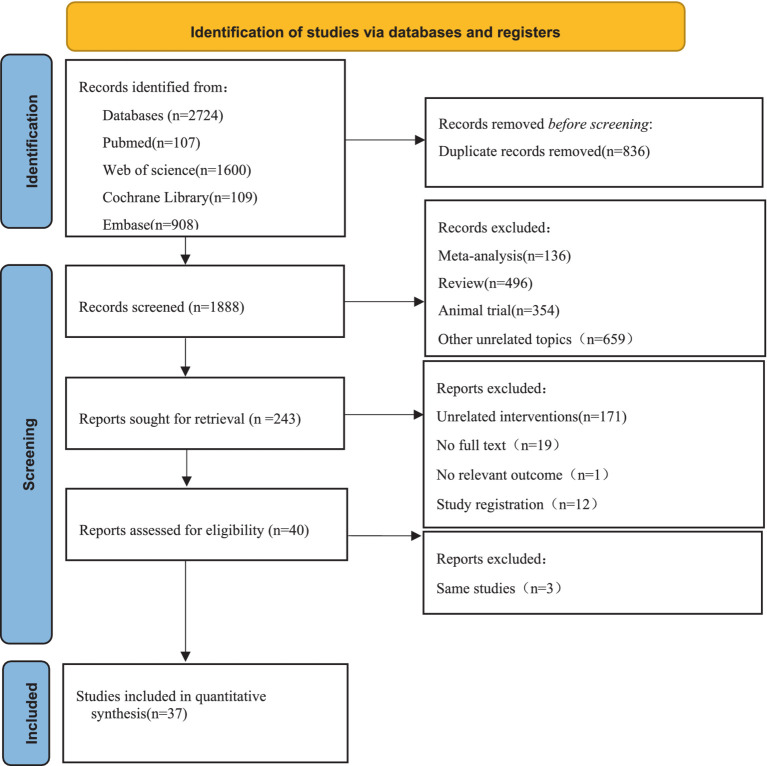
Flow diagram of the search strategy and study selection.

According to the pre-determined retrieval strategy, 2,724 documents were retrieved from Pubmed, Web of science, Embase, and Cochrane library. 836 duplicated articles were removed, and the documents that did not meet the criteria were excluded by reading the abstract and full text of the documents. Finally, 37 articles were included. The specific retrieval process was shown in Figure 1.

### 7.2 Characteristics of included studies

The 37 studies included 1921 patients, including 266 pediatrics and 1,655 adults. The articles were published from 2011 to 2024, and 35 of them are double-arm studies, one of them is three-arm study, and one of them is four-arm study. 5 MTTs were included: probiotics (23 RCTs), synbiotics (10 RCTs), prebiotics (4 RCTs), FMT (3 RCTs), antibiotics (1 RCT). More details of included studies were shown in Table 1.

**Table 1:** Characteristics of included studies.

Study ID	Diagnosis	Patient population	Diagnostic criteria	Sample size (M/F)	Age (year)	Intervention experimental group	Control group	Duration (week)	Outcomes
Xue et al. (42)	NAFLD	Adult	(AASL) and Treatment of Non-Alcoholic Fatty Liver Disease (2018 revision)	75(39/36)	E:(57.3 ± 13.4) C:(60.2 ± 8.5)	Allogenic FMT	probiotics	4	BMI HOMA-IR UA AST ALT TBIL ALB TC TG LDL-C HDL-C LSM
Wong et al. (43)	NASH	Adult	Histology-proven	20(13/7)	E:(42 ± 9) C:(55 ± 9)	Probiotics and prebiotics	Usual care group	24	BMI ALT AST HDL-C LDL-C LSM Triglycerides
Witjes et al. (44)	NAFLD	Adult	Ultrasound	21(19/2)	E:(51.2 ± 6.6) C:(48.5 ± 10.2)	Allogenic FMT,	Autologous FMT^1^	24	GGT AST ALT Cholesterol HDL-C LDL-C
Vajro et al. (19)	NAFLD	Pediatric	Ultrasound &ALT	20(−/−)	/	Lactobacillus GG	Placebo	8	ALT BMI
Sepideh et al. (45)	NAFLD	Adult	Ultrasound	42(28/14)	E:(42.10 ± 1.99) C:(47.33 ± 2.53)	Probiotics	Placebo	8	HOMA-IR
Scorletti et al. (46)	NAFLD	Adult	/	89(58/31)	E:50.2(12.4) C:51.6(13.1)	Synbiotics	Placebo	48	BMI TC HDL-C LDL-C Triglycerides ALT AST GGT LSM CAP
Rodrigo et al. (47)	NAFLD/NASH	Pediatric	Ultrasound & AST/ALT ratio < 1	84(62/22)	E:(11.28 ± 1.87) C:(12.05 ± 1.45)	Probiotics	Placebo	24	AST ALT CAP LSM BMI TC TG HDL LDL GGT
Nor et al. (48)	NAFLD	Adult	Ultrasound& CAP& ALT	39(28/11)	E:54.70(10.19) C:52.47(16.73)	Probiotics	Placebo	24	CAP TC TG ALT AST GGT LSM BMI
Mofidi et al. (49)	NAFLD	Adult	CAP &ALT	42(23/19)	E:(40.09 ± 11.44) C:(44.61 ± 10.12)	Synbiotics	Placebo	28	CAP LSM AST ALT HOMA-IR HDL-C LDL-C TC
Manzhalii et al. (50)	NASH	Adult	Ultrasound &ALT	75(27/48)	E:(44.3 ± 1.5) C:(43.5 ± 1.3)	Probiotics	Usual care	12	BMI TC TG ALT AST GGT LSM
Malaguarnera et al. (51)	NASH	Adult	Ultrasound &liver biopsy &ALT	66(33/33)	E:(46.9 ± 5.4) C:(46.7 ± 55.7)	Synbiotics	Placebo	24	BMI AST ALT TC TG HDL-C LDL-C HOMA-IR
Javadi et al. (20)	NAFLD	Adult	Ultrasound ALT	75(60/15)	E:(43.9 ± 9.02); (38.7 ± 10); (43.2 ± 6.95); C:(42.2 ± 9.11)	Probiotics\prebiotics\synbiotics	Placebo	12	BMI TC TG HDL-C LDL-C HOMA-IR
Goyal et al. (52)	NAFLD	Pediatrics	Ultrasound	54(/)	E:(11.7 ± 2.21) C:(11.0 ± 1.20)	VSL#3	Placebo	16	BMI AST ALT GGT LDL-C HDL-C Cholesterol
Ferolla et al. (53)	NASH	Adult	liver biopsy	50(12/38)	/	Synbiotics	Usual care	12	BMI Cholesterol HDL-C LDL-C Triglycerides ALT AST
Famouri et al. (54)	NAFLD	Pediatric	Ultrasound	64(32/32)	E:12.7(2.2) C:12.6(1.7)	Probiotics	Placebo	12	AST ALT HDL-C LDL-C BMI Cholesterol Triglyceride
Eslamparast et al. (55)	NAFLD	Adult	Ultrasound	52(25/27)	E:(46.35 ± 8.8) C:(45.69 ± 9.5)	Synbiotics	Placebo	28	BMI ALT AST HOMA-IR LSM
Duseja et al. (56)	NAFLD	Adult	ALT& AST	39(28/11)	E:38(10) C:33(6)	Probiotics	Placebo	48	BMI AST BIL ALT
Craven et al. (57)	NAFLD	Adult	AASLD	21(6/15)	E:47.6(14.9) C:57.5(13.0)	Allogenic FMT	Autologous FMT	24	BMI
Chong et al. (58)	NAFLD	Adult	/	35(28/7)	E:(57 ± 8) C:(58 ± 7)	VSL#3	Placebo	10	TC HDL LDL Triglycerides HOMA-IR ALT AST BMI
Cai et al. (59)	NAFLD	Adult	Ultrasound &liver biopsy &ALT	140(85/55)	E:(46.13 ± 12.72) C:(49.62 ± 9.08)	Probiotics	Usual care	12	ALT TBIL AST TG TC LDL-C HDL-C HOMA-IR
Bomhof et al. (60)	NASH	Adult	liver biopsy	14(8/6)	E:(45.3 ± 5.6) C:(53.5 ± 4.8)	Prebiotics	Placebo	24	BMI ALT HOMA-IR
Behrouz et al. (61)	NAFLD	Adult	ALT& Ultrasound	89(63/26)	E1:(38.46 ± 7.11) E2:(38.41 ± 9.21) C:(38.43 ± 10.09)	Probiotics/prebiotics	Placebo	12	BMI TG TC HDL-C LDL-C ALT AST Triglyceride
Asgharian et al. (62)	NAFLD	Adult	Ultrasound	74(19/55)	E:(46.57 ± 1.7) C:(47.78 ± 1.7)	Synbiotics	Placebo	8	ALT AST BMI
Aller et al. (63)	NAFLD	Adult	liver biopsy	28(14/14)	E:(49.4 ± 10.9) C:(44.3 ± 15.1)	Probiotics	Placebo	12	BMI TC LDL-C HDL-C TG HOMA-IR
Alisi et al. (64)	NAFLD	Pediatric	biopsy-proven	44(24/20)	E:10(9,12) C:11(10,12)	VSL3#	Placebo	16	Triglycerides HOMA-IR BMI ALT
Ahn et al. (65)	NAFLD	Adult	/	65(33/32)	E:(41.7 ± 12.49) C:(44.71 ± 13.31)	Probiotics	Placebo	12	BMI CAP LSM Cholesterol Triglyceride AST ALT
Abhari et al. (66)	NAFLD	Adult	CAP &ALT	45(25/20)	E:(47.7 ± 11.4) C:(46.7 ± 12.4)	Synbiotics	Placebo	12	CAP AST ALT BMI Cholesterol TG TC LDL-C HDL-C HOMA-IR
Sayari et al. (67)	NAFLD	Adult	ALT& Ultrasound	140(85/55)	E:(42.48 ± 11.41) C:(43.42 ± 11.65)	Synbiotics+ sitagliptin	Placebo (sitagliptin in both groups)	16	BMI TC ALT AST TG LDL HDL
Sadrkabir et al. (68)	NAFLD	Adult	Ultrasound	61(40/21)	E:(43.26 ± 11.42) C:(43.72 ± 10.76)	Gerilact	Placebo	8	BMI AST ALT TG LDL HDL CHOL
Kobyliak et al. (69)	NAFLD	Adult	AASLD	58(−/−)	E:(53.4 ± 9.55) C:(57.29 ± 10.45)	Probiotics	Placebo	8	BMI LSM ALT AST GGT TC TG HDL-C LDL-C
Ekhlasi et al. (70)	NAFLD	Adult	ALT &Ultrasound	30(−/−)	/	Synbiotics	Placebo	8	BMI ALT AST
Abdel-Razik et al. (71)	NASH	Adult	Liver biopsy-proven	50(16/34)	E:(40.2 ± 9.88) C:(38.4 ± 9.21)	Rifaximin	Placebo	24	ALT AST HOMA-IR BMI
Ayob et al. (38)	NAFLD	Adult	ALT &Ultrasound	40(29/11)	E:(55 ± 11.07) C:(49.95 ± 14.05)	Probiotics	Placebo	24	BMI ALT AST TG TC
Derosa et al. (72)	NAFLD	Adult	ALT	60(28/32)	E:(55.8 ± 7) C:(56.76 ± 7.7)	VASL#3	PLACEBO	12	BMI TC LDL-C HDL-C TG AST ALT
Escouto et al. (73)	NASH	Adult	Liver biopsy-proven	48(10/38)	E:(58 ± 31.85) C:(57 ± 28.15)	probiotics	Placebo	24	BMI ALT AST TC HDL-C LDL-C TG HOMA-IR
Reshef et al. (74)	NAFLD	Adult	Ultrasound	50(15/35)	E:(45.72 ± 8.9) C:(46.48 ± 11.6)	probiotics	UC	12	AST ALT
Naama et al. (75)	NAFLD	Adult	Ultrasound & ALT	19(15/4)	C:(50 ± 14.52) E:(47.8 ± 10.37)	Prebiotics	Placebo	12	BMI HOMA-IR TC HDL-C LDL-C TG ALT AST

“/” represents that it is not mentioned in the text; E: Experimental group; C: Control group.

### 7.3 Risk of bias

**Figure 2:**
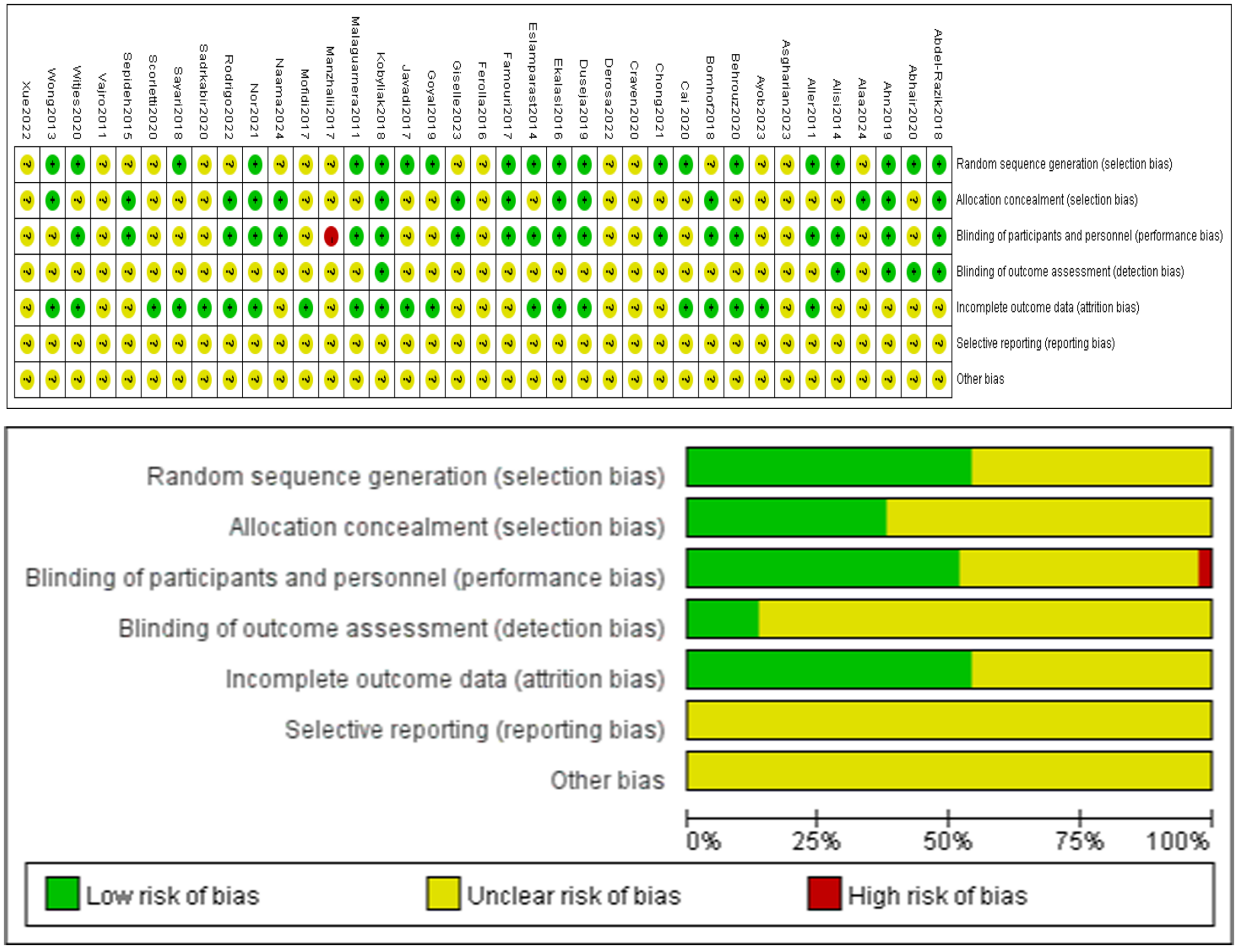
Risk of bias.

Review Manager 5.3 was used to draw a Cochrane bias risk. The risk assessment of 37 RCTs was shown in Figure 2. 20 studies used appropriate randomization methods, such as computer-generated random numbers or tables. 14 studies indicated allocation concealment, such as treatment allocation being packed in identical packages, sachets or same envelope. One non-blind study (19) was evaluated as “high risk.” 20 studies provided detailed descriptions of data integrity, including records of missing data and retention status at different stages. These were rated as “low risk.” None of the studies indicated selective reporting and were rated “unclear.” The result of risk assessment was shown in Figure 2.

### 7.4 Network meta-analysis

#### 7.4.1 Primary outcome

**Figure 3:**
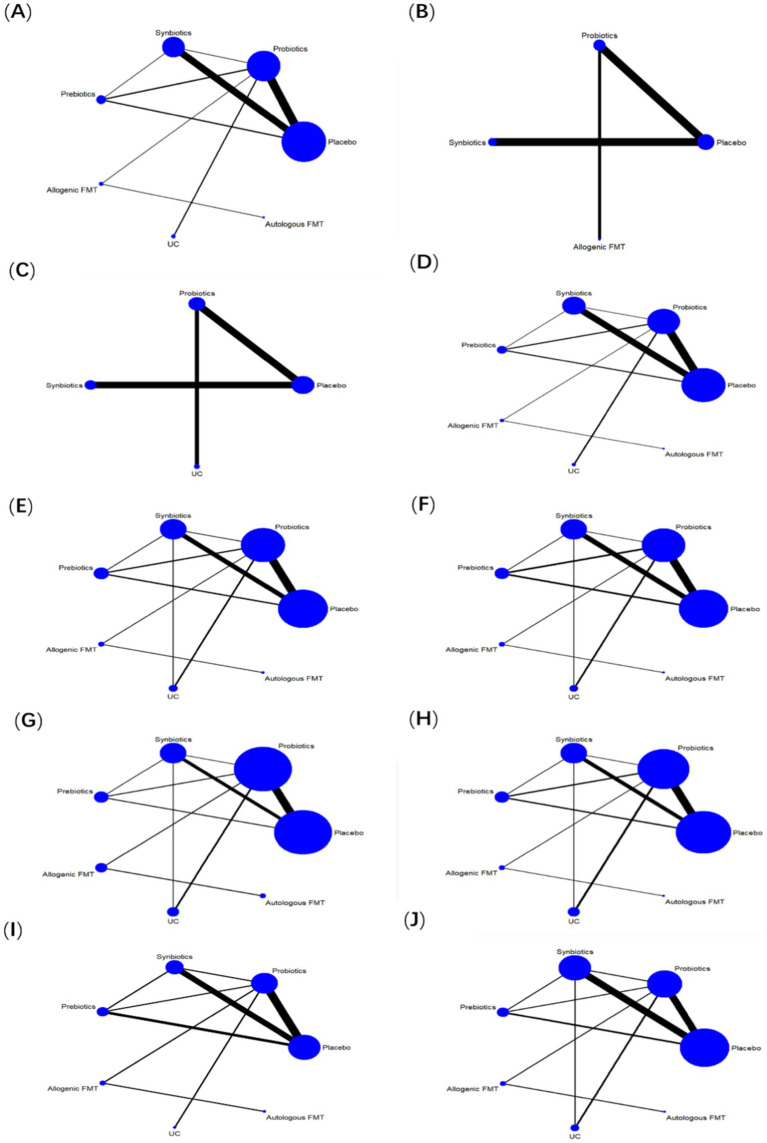
Network evidence plots. **(A)** ALT **(B)** CAP **(C)** LSM **(D)** AST **(E)** HDL-C **(F)** LDL-C **(G)** TG **(H)** TC **(I)** HOMA-IR **(J)** BMI.

**Figure 4:**
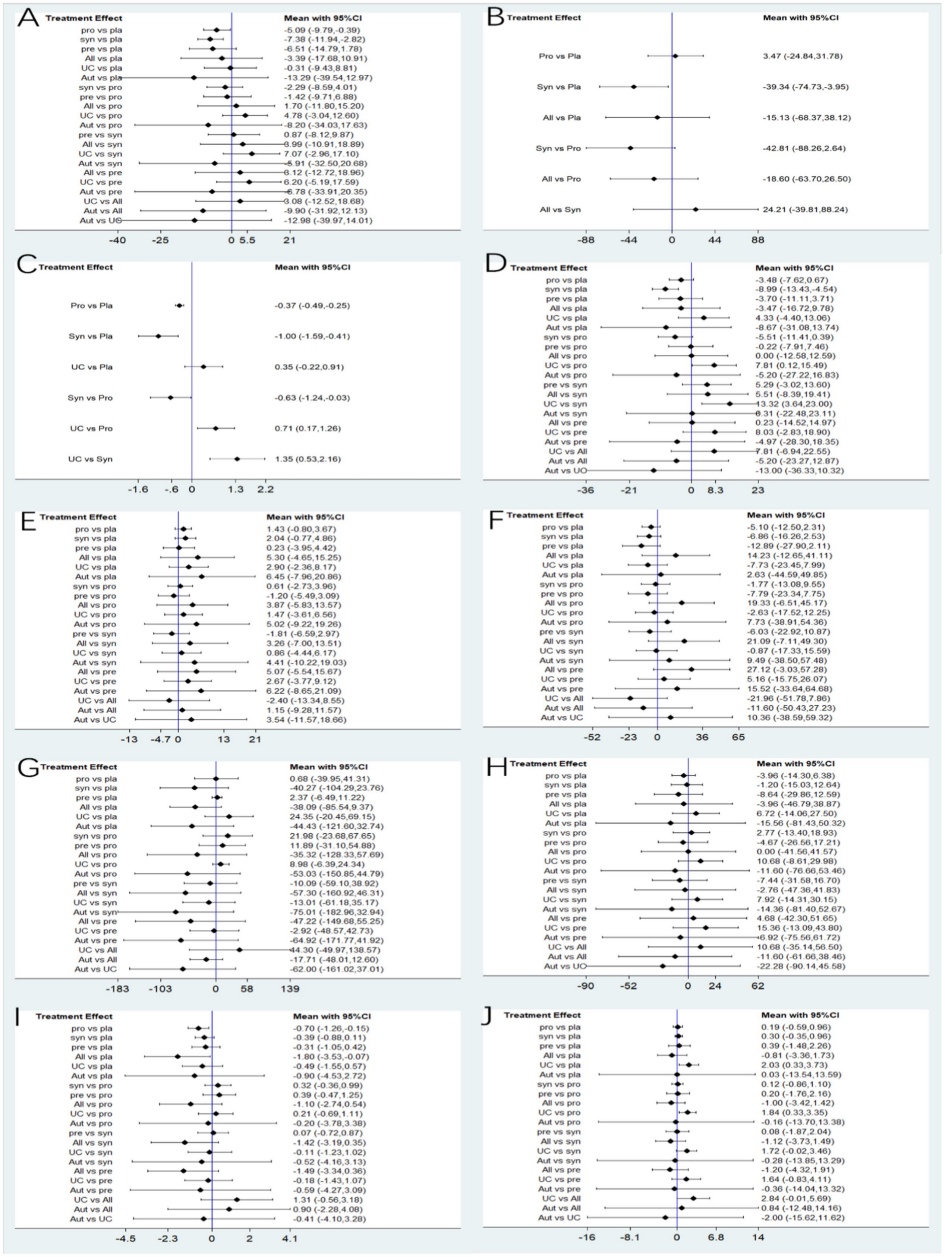
Forest plots. **(A)** ALT **(B)** CAP **(C)** LSM **(D)** AST **(E)** HDL-C **(F)** LDL-C **(G)** TG **(H)** TC **(I)** HOMA-IR **(J)** BMI.

**Figure 5:**
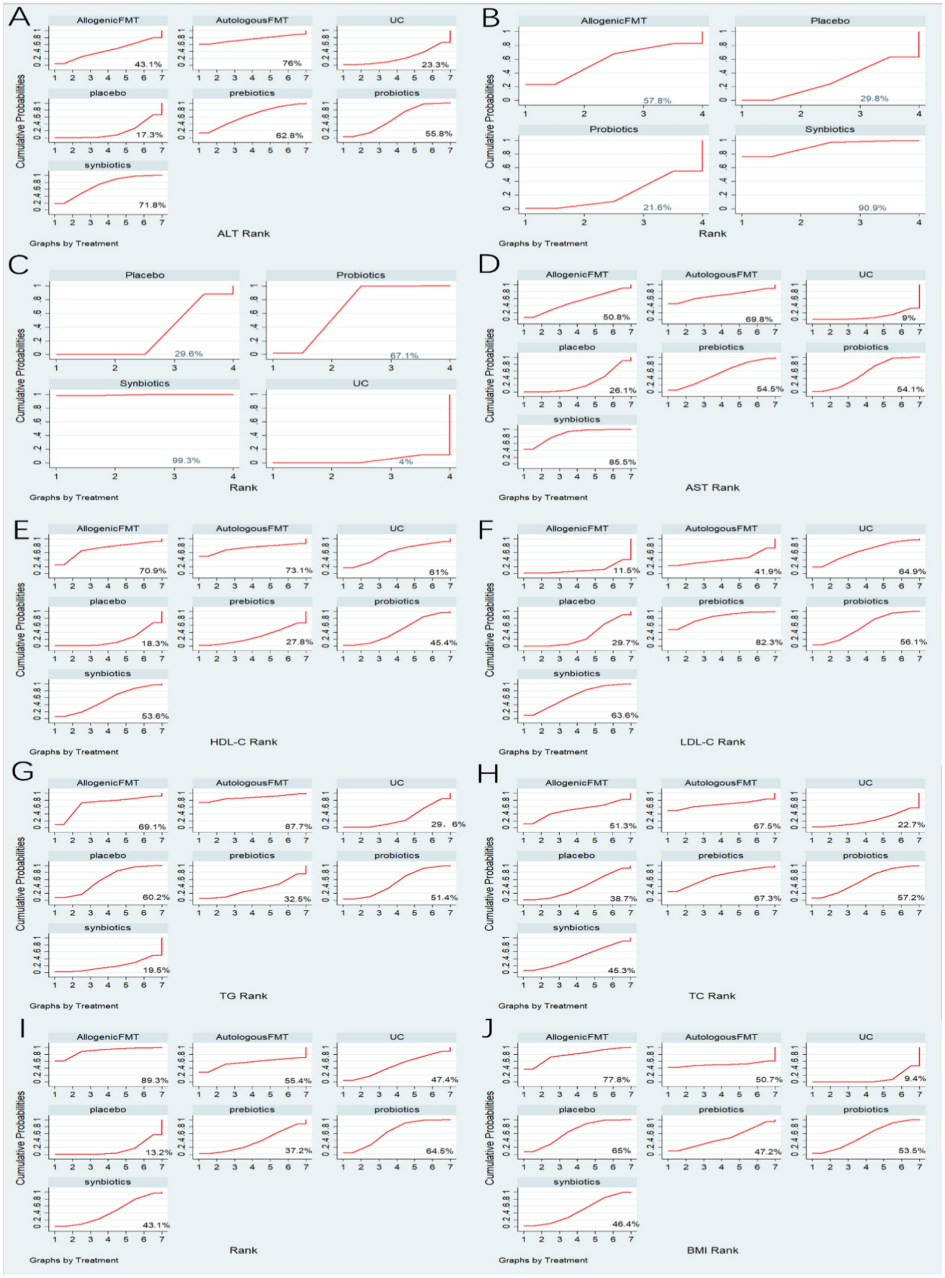
Summary of results from SUCRA. **(A)** ALT **(B)** CAP **(C)** LSM **(D)** AST **(E)** HDL-C **(F)** LDL-C **(G)** TG **(H)** TC **(I)** HOMA-IR **(J)** BMI.

30 studies reported ALT, involving probiotics, prebiotics, synbiotics, FMT, of which the network relationship between the interventions is shown in Figure 3. In terms of ALT improvement, according to MD and 95%CI between all the pairwise interventions, probiotics (MD: −5.09; 95%CI: −9.79, −0.39), synbiotics (MD: −7.38; 95CI%: −11.94, −2.82) were superior to placebo. As shown in Table 2 and Figure 4. In addition, Autologous FMT, with the highest-ranking probability of SUCRA (76%), had the best effectiveness in reducing ALT, followed by prebiotics (62.8%) and synbiotics (71.8%). More details about the rank probability of SUCRA are shown in Figure 5.

**Table 2:** The league table of ALT and CAP.

Autologous FMT	-	-	-	-	-	-
−5.91 (−32.50, 20.68)	Synbiotics	-	−42.81 (−88.26, 2.64)	−24.21 (−88.24, 39.81)	-	**−39.34 (−74.73, −3.95)**
−6.78 (−33.91, 20.35)	−0.87 (−9.87, 8.12)	Prebiotics	-	-	-	-
−8.20 (−34.03, 17.63)	−2.29 (−8.59, 4.01)	−1.42 (−9.71, 6.88)	Probiotics	−18.60 (−63.70, 26.50)	-	−3.47 (−31.78, 24.84)
−9.90 (−31.92, 12.13)	−3.99 (−18.89, 10.91)	−3.12 (−18.96, 12.72)	−1.70 (−15.20, 11.80)	Allogenic FMT	-	−15.13 (−68.37, 38.12)
−12.98 (−39.97, 14.01)	−7.07 (−17.10, 2.96)	−6.20 (−17.59, 5.19)	−4.78 (−12.60, 3.04)	−3.08 (−18.68, 12.52)	UC	-
−13.29 (−39.54, 12.97)	−7.38 (−11.94, −2.82)	−6.51 (−14.79, 1.78)	−5.09 (−9.79, −0.39)	−3.39 (−17.68, 10.91)	−0.31 (−9.43, 8.81)	Placebo

Comparisons for ALT (bottom left) and CAP (upper right) of MTTs. Data of comparison for ALT and CAP, are MD(95% CI). The 95% CI confidence interval which does not range across 0 favors the column-defining treatment and is shown in bold.

7 studies reported CAP, involving probiotics, synbiotics, Allogenic FMT, of which the network relationship between the interventions is shown in Figure 3. In terms of CAP improvement, according to MD and 95%CI between all pairwise interventions, synbiotics (MD: −39.34; 95%CI: −74.73, −3.95) was superior to placebo. As shown in Table 2 and Figure 4. In addition, synbiotics (90.9%), had the best effectiveness in reducing CAP, followed by Alllogenic FMT (57.8%). More details about the rank probability of SUCRA are shown in Figure 5.

10 studies reported LSM, involving probiotics, synbiotics, of which the network relationship between the interventions is shown in Figure 3. In terms of LSM improvement, according to MD and 95%CI between all pairwise interventions, synbiotics (MD: −1.00; 95%CI: −1.59, −0.41), probiotics (MD: −0.37; 95%CI: −0.49, −0.25) was superior to placebo. Moreover, synbiotics (MD: −1.35; 95%CI: −12.16, −0.53), probiotics (MD: −0.71; 95%CI: −1.26, −0.17) was superior to UC. As shown in Table 3 and Figure 4. In addition, synbiotics (MD: −0.63; 95%CI: −1.24, −0.03) was better than probiotics. According to SUCRA, synbiotics (99.3%), had the best effectiveness in reducing LSM, followed by probiotics (67.1%). More details about the rank probability of SUCRA are shown in Figure 5.

**Table 3:** The league table of AST and LSM.

Synbiotics	-	-	**−0.63 (−1.24, −0.03)**		**−1.00 (−1.59, −0.41)**	**−1.35 (−2.16, −0.53)**
−0.31 (−23.11, 22.48)	Autologous FMT	-	-	-	-	-
−5.29 (−13.60, 3.02)	−4.97 (−28.30, 18.35)	Prebiotics	-	-	-	-
−5.51 (−11.41, 0.39)	−5.20 (−27.22, 16.83)	−0.22 (−7.91, 7.46)	Probiotics	-	**−0.37 (−0.49, −0.25)**	**−0.71 (−1.26, −0.17)**
−5.51 (−19.41, 8.39)	−5.20 (−23.27, 12.87)	−0.23 (−14.97, 14.52)	−0.00 (−12.59, 12.58)	Allogenic FMT	-	-
−8.99 (−13.43, −4.54)	−8.67 (−31.08, 13.74)	−3.70 (−11.11, 3.71)	−3.48 (−7.62, 0.67)	−3.47 (−16.72, 9.78)	Placebo	−0.35 (−0.91, 0.22)
−13.32 (−23.00, −3.64)	−13.00 (−36.33, 10.32)	−8.03 (−18.90, 2.83)	−7.81 (−15.49, −0.12)	−7.81 (−22.55, 6.94)	−4.33 (−13.06, 4.40)	UC

Comparison for AST (bottom left) and LSM (upper right) of MTTs. Data of comparison for AST and LSM, are MD (95% CI). The 95%CI confidence interval which does not range across 0 favors the column-defining treatment and is shown in bold.

#### 7.4.2 Secondary outcome

27 studies reported AST, involving probiotics, synbiotics, probiotics, Allogenic FMT, Autologous FMT. The network relationship between the interventions is shown in Figure 3. In terms of AST improvement, synbiotics (MD: −8.99; 95%CI: −13.43, −4.54) was superior to placebo. Moreover, synbiotics (MD: −13.32; 95%CI: −23, −3.64), and probiotics (MD: −7.81; 95%CI: −15.49, −0.12) were better than UC as shown in Table 3 and Figure 4. According to SUCRA, synbiotics (85.5%), had the best effectiveness in reducing AST, followed by Autologous FMT (69.8%), prebiotics (54.5%). More details about the rank probability of SUCRA are shown in Figure 5.

21 studies reported HDL-C, involving probiotics, synbiotics, probiotics, Allogenic FMT, Autologous FMT. The network relationship between the interventions is shown in Figure 3. In terms of HDL-C improvement, there was no significant difference between all interventions and placebo, and there was no difference between 5 interventions as shown in Table 4 and Figure 4. However, Autologous FMT (73.1%), Allogenic FMT (70.9%) with highest-ranking probability of SUCRA, had the best effectiveness in reducing HDL-C. More details about the rank probability of SUCRA are shown in Figure 5.

**Table 4:** The league table of HDL and LDL.

Allogenic FMT	−14.23 (−41.11, 12.65)	−19.33 (−45.17, 6.51)	−11.60 (−50.43, 27.23)	−8.71 (−57.91, 40.48)	−21.09 (−49.30, 7.11)	−21.96 (−51.78, 7.86)
5.30 (−4.65, 15.25)	Placebo	−5.10 (−12.50, 2.31)	2.63 (−44.59, 49.85)	−20.31 (−49.69, 9.06)	−6.86 (−16.26, 2.53)	−7.73 (−23.45, 7.99)
3.87 (−5.83, 13.57)	−1.43 (−3.67, 0.80)	Probiotics	−7.73 (−54.36, 38.91)	−1.45 (−18.68, 15.79)	−1.77 (−13.08, 9.55)	−2.63 (−17.52, 12.25)
−1.15 (−11.57, 9.28)	−6.45 (−20.86, 7.96)	−5.02 (−19.26, 9.22)	AutologousFMT	−0.99 (−13.04, 11.06)	−9.49 (−57.48, 38.50)	−10.36 (−59.32, 38.59)
5.07 (−5.54, 15.67)	−0.23 (−4.42, 3.95)	1.20 (−3.09, 5.49)	6.22 (−8.65, 21.09)	Prebiotics	−4.69 (−23.10, 13.72)	−6.72 (−16.53, 3.09)
3.26 (−7.00, 13.51)	−2.04 (−4.86, 0.77)	−0.61 (−3.96, 2.73)	4.41 (−10.22, 19.03)	−1.81 (−6.59, 2.97)	Synbiotics	−0.87 (−17.33, 15.59)
2.40 (−8.55, 13.34)	−2.90 (−8.17, 2.36)	−1.47 (−6.56, 3.61)	3.54 (−11.57, 18.66)	−2.67 (−9.12, 3.77)	−0.86 (−6.17, 4.44)	UC

Comparisons for HDL (bottom left) and LDL (upper right) of MTTs.

21 studies reported LDL-C, involving probiotics, synbiotics, probiotics, Allogenic FMT, Autologous FMT. The network relationship between the interventions is shown in Figure 3. In terms of LDL-C improvement, there was no significant difference between all interventions and placebo, and there was no difference between 5 interventions as shown in Table 4 and Figure 4. However, prebiotics (82.3%) with highest-ranking probability of SUCRA, had the best effectiveness in reducing LDL-C. More details about the rank probability of SUCRA are shown in Figure 5.

27 studies reported TG, involving probiotics, synbiotics, probiotics, Allogenic FMT, Autologous FMT. The network relationship between the interventions is shown in Figure 3. There was no significant difference between all interventions and placebo, and there was no difference between 5 interventions as shown in Table 5 and Figure 4. However, Autologous FMT (87.7%) with highest-ranking probability of SUCRA, had the best effectiveness in reducing TG, followed by Allogenic FMT (69.1%). More details about the rank probability of SUCRA are shown in Figure 5.

**Table 5:** The league table of TG and TC.

Autologous FMT	−11.60 (−61.66, 38.46)	−15.56 (−81.43, 50.32)	−11.60 (−76.66, 53.46)	−6.92 (−75.56, 61.72)	−22.28 (−90.14, 45.58)	−14.36 (−81.40, 52.67)
−17.71 (−48.01, 12.60)	Allogenic FMT	−3.96 (−46.79, 38.87)	−0.00 (−41.57, 41.56)	−4.68 (−51.65, 42.30)	−10.68 (−56.50, 35.14)	−2.76 (−47.36, 41.83)
−44.43 (−121.60, 32.74)	−38.09 (−85.54, 9.37)	Placebo	−3.96 (−14.30, 6.38)	−8.64 (−29.86, 12.59)	−6.72 (−27.50, 14.06)	−1.20 (−15.03, 12.64)
−53.03 (−150.85, 44.79)	−35.32 (−128.33, 57.69)	−0.68 (−41.31, 39.95)	Probiotics	−4.67 (−26.56, 17.21)	−10.68 (−29.98, 8.61)	−2.77 (−18.93, 13.40)
−64.92 (−171.77, 41.92)	−47.22 (−149.68, 55.25)	−2.37 (−11.22, 6.49)	−11.89 (−54.88, 31.10)	Prebiotics	−15.36 (−43.80, 13.09)	−7.44 (−31.58, 16.70)
−62.00 (−161.02, 37.01)	−44.30 (−138.57, 49.97)	−24.35 (−69.15, 20.45)	−8.98 (−24.34, 6.39)	2.92 (−42.73, 48.57)	UC	−7.92 (−30.15, 14.31)
−75.01 (−182.96, 32.94)	−57.30 (−160.92, 46.31)	40.27 (−23.76, 104.29)	−21.98 (−67.65, 23.68)	−10.09 (−59.10, 38.92)	−13.01 (−61.18, 35.17)	Synbiotics

Comparisons for TG (bottom left) and TC (upper right) of MTTs.

24 studies reported TC, involving probiotics, synbiotics, prebiotics, FMT. The network relationship between the interventions is shown in Figure 3. There was no significant difference between all interventions and placebo, and there was no difference between 5 interventions as shown in Table 5 and Figure 4. However, Autologous FMT (67.5%) with highest-ranking probability of SUCRA, had the best effectiveness in reducing TC, followed by prebiotics (67.3%) and probiotics (57.2%) More details about the rank probability of SUCRA are shown in Figure 5.

14 studies reported HOMA-IR, involving probiotics, synbiotics, probiotics, Allogenic FMT, Autologous FMT. The network relationship between the interventions is shown in Figure 3. Probiotics (MD: −0.7, 95%CI: −1.26, −0.15) and Allogenic FMT (MD: −1.8, 95%CI: −3.53, − 0.07) was better than placebo. Moreover, Allogenic FMT (89.3%) with highest-ranking probability of SUCRA, had the best effectiveness in reducing HOMA-IR, followed by probiotics (64.5%) and Autologous FMT (55.4%). More details about the rank probability of SUCRA are shown in Figure 5.

22 studies reported BMI, involving probiotics, synbiotics, probiotics, Allogenic FMT, Autologous FMT. The network relationship between the interventions is shown in Figure 3. Probiotics (MD: −1.84, 95%CI: −3.35, −0.33) was better than UC as shown in Table 6 and Figure 4. However, Allogenic FMT (77.8%) with highest-ranking probability of SUCRA, had the best effectiveness in reducing BMI, followed by placebo (65%). More details about the rank probability of SUCRA are shown in Figure 5.

**Table 6:** The league table of HOMA-IR and BMI.

Allogenic FMT	−1.00 (−3.42, 1.42)	−0.84 (−14.16, 12.48)	−2.84 (−5.69, 0.01)	−1.12 (−3.73, 1.49)	−1.20 (−4.32, 1.91)	−0.81 (−3.36, 1.73)
−1.10 (−2.74, 0.54)	Probiotics	0.16 (−13.38, 13.70)	**−1.84 (−3.35, −0.33)**	−0.12 (−1.10, 0.86)	−0.20 (−2.16, 1.76)	−0.19 (−0.96, 0.59)
−0.90 (−4.08, 2.28)	0.20 (−3.38, 3.78)	Autologous FMT	−2.00 (−15.62, 11.62)	−0.28 (−13.85, 13.29)	−0.36 (−14.04, 13.32)	−0.03 (−13.59, 13.54)
−1.31 (−3.18, 0.56)	−0.21 (−1.11, 0.69)	−0.41 (−4.10, 3.28)	UC	−1.72 (−3.46, 0.02)	−1.64 (−4.11, 0.83)	−2.03 (−3.73, −0.33)
−1.42 (−3.19, 0.35)	−0.32 (−0.99, 0.36)	−0.52 (−4.16, 3.13)	−0.11 (−1.23, 1.02)	Synbiotics	0.08 (−1.87, 2.04)	−0.30 (−0.96, 0.35)
−1.49 (−3.34, 0.36)	−0.39 (−1.25, 0.47)	−0.59 (−4.27, 3.09)	−0.18 (−1.43, 1.07)	−0.07 (−0.87, 0.72)	Prebiotics	−0.39 (−2.26, 1.48)
**−1.80 (−3.53, −0.07)**	**−0.70 (−1.26, −0.15)**	−0.90 (−4.53, 2.72)	−0.49 (−1.55, 0.57)	−0.39 (−0.88, 0.11)	−0.31 (−1.05, 0.42)	Placebo

Comparisons for HOMA-IR (bottom left) and BMI (upper right) of MTTs. Data of comparison for HOMA-IR and BMI, are MD (95% CI). The 95% CI confidence interval which does not range across 0 favors the column-defining treatment and is shown in bold.

### 7.5 Adverse reaction

6 studies reported specific adverse reactions. Of these, two studies focused on the adverse effects of probiotics, mainly including symptoms of the digestive system such as diarrhea, flatulence, nausea, and other symptoms like mild headache, urinary tract infection, and adperianalrash. There was no difference in the incidence of adverse reactions in the digestive system between the probiotics group and placebo group (*p* = 0.857); two studies focused on the adverse effects of synbiotics, including moderate headaches, nausea, abdominal pain. There was no difference in the incidence of adverse reactions in the digestive system between the synbiotics group and placebo group (*p* = 0.62); one study reported the occurrence of flatulence in both the prebiotics group and placebo group. One study reported the adverse reactions of antibiotics, which were confined to the gastrointestinal adverse events, specifically comprising abdominal pain, nausea, vomiting, constipation and diarrhea. No significant difference was observed in the incidence rates between the intervention group and the control group. More details about adverse reactions are shown in Table 7 and Supplementary Table S2.

**Table 7:** Adverse reaction.

Study ID	Intervention of experiment group	Intervention of control group	Adverse reactions in experiment group	Adverse reactions in control group
Abhari et al. (66)	Synbiotics	Placebo	*N*	*N*
Ayob et al. (38)	Probiotics	Placebo	*N*	*N*
Ahn et al. (65)	Probiotics	Placebo	*N*	*N*
Alisi et al. (64)	VSL3#	Placebo	*N*	*N*
Aller et al. (63)	Probiotics	Placebo	*N*	*N*
Asgharian et al. (62)	Synbiotics	Placebo	*N*	*N*
Behrouz et al. (61)	Prebiotics/probrotic	Placebo	*N*	*N*
Bomhof et al. (60)	Oligofructose	Placebo	Flatulence (*n* = 1)	Flatulence (*n* = 1)
Cai et al. (59)	Probiotics	Usual Care	*N*	*N*
Chong et al. (58)	VSL#3	Placebo	Urinary tract infection (*n* = 3); Bloating (*n* = 2); Nausea (*n* = 2); Genital thrush (*n* = 1); Adperianalrash (*n* = 1).	Diarrhea (*n* = 1), Abdominal Cramps (*n* = 1); Back pain (n = 1); Traumatic toe infection (*n* = 1)
Craven et al. (57)	Allogenic FMT	Autologous FMT	*N*	*N*
Derosa et al. (72)	VSL#3	Placebo	*N*	*N*
Duseja et al. (56)	Probiotics	Placebo	*N*	*N*
Ekalasi et al. (70)	Synbiotics	Placebo	*N*	*N*
Eslamparast et al. (55)	Synbiotics	Placebo	Moderate headaches (*n* = 1)	Abdominal pain (*n* = 1)
Famouri et al. (54)	Probiotics	Placebo	*N*	*N*
Ferolla et al. (53)	Synbiotics	Placebo	*N*	*N*
Goyal et al. (52)	VSL#3	Placebo	*N*	*N*
Javadi et al. (20)	Probiotics/prebiotics/synbiotics	Placebo	*N*	*N*
Kobyliak et al. (69)	Probiotics	Placebo	Short-term Diarrhea (*n* = 1); Mild headaches (*n* = 1)	Mild abdominal pain (*n* = 2); Nausea (*n* = 1).
Malaguarnera et al. (51)	Synbiotics	Placebo	Nausea (*n* = 1); Moderate headache (*n* = 1); Abdominal pain (*n* = 1).	Nausea (*n* = 2); Fatigue (*n* = 1); Dizziness (*n* = 1)
Manzhalii et al. (50)	Probiotics	Placebo	*N*	*N*
Mofidi et al. (49)	Synbiotics	Placebo	*N*	*N*
Nor et al. (48)	Probiotics	Placebo	*N*	*N*
Rodrigo et al. (47)	Probiotics	Placebo	*N*	*N*
Sadrkabir et al. (68)	Synbiotics	Placebo	*N*	*N*
Sayari et al. (67)	Sitagliptin-synbiotics	Sitagliptin-placebo	*N*	*N*
Scorletti et al. (46)	Synbiotics	Placebo	*N*	*N*
Sepiden et al. (45)	Probiotics	Placebo	*N*	*N*
Vajro et al. (19)	Probiotics	Placebo	*N*	*N*
Witjes et al. (44)	Allogenic FMT	Autologous FMT	*N*	*N*
Wong et al. (43)	Probiotics and prebiotics	Usual Care	*N*	*N*
Xue et al. (42)	Allogenic FMT	Probiotics	*N*	*N*
Abdel-Razik et al. (71)	Rifaximin	Placebo	Abdominal pain (*n* = 1) Nausea (*n* = 2) Vomiting (*n* = 1) Constipation (*n* = 1) Diarrhea (*n* = 2)	Abdominal pain (*n* = 2) Nausea (*n* = 2) Vomiting (*n* = 1) Constipation (*n* = 1) Diarrhea (*n* = 1)
Giselle et al. (73)	Probiotics	Placebo	*N*	*N*
Reshef et al. (74)	Probiotics	UC	*N*	*N*
Naama et al. (75)	Prebiotics	Placebo	*N*	*N*

### 7.6 Network inconsistency and publication bias

**Figure 6:**
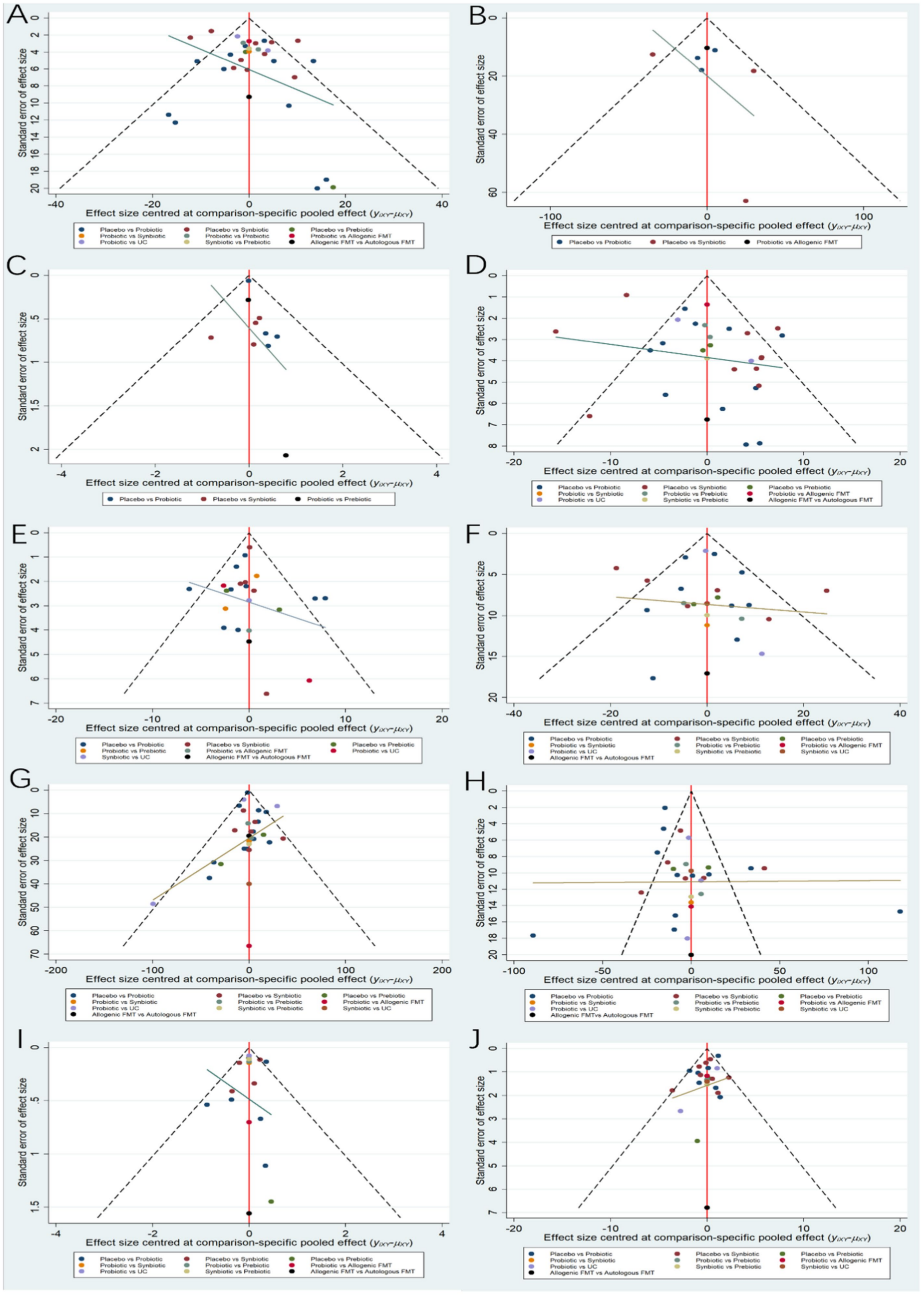
Funnel plots. **(A)** ALT **(B)** CAP **(C)** LSM **(D)** AST **(E)** HDL-C **(F)** LDL-C **(G)** TG **(H)** TC **(I)** HOMA-IR **(J)** BMI.

The network evidence diagrams of ALT, AST, TG, TC, HDL-C, LDL-C, BMI and HOMA-IR formed a closed loop, respectively. Network inconsistency was used to evaluate the inconsistency. The results of global-inconsistency assessment are shown in Supplementary Table S2. There were no evidences of inconsistency in the indicators ALT, AST, TG, TC, LDL-C, HDL-C and BMI. However, the *p*-value for test of global inconsistency of HOMA-IR is significant (*p* = 0.0006). The node-splitting method was performed to evaluate the inconsistency. The results of inconsistency showed that there was inconsistency in the comparison between prebiotics and placebo, as well as those between probiotics and prebiotics. Subsequently, leave-one-out method was used to remove the study (20) it was demonstrated that the inconsistency was not significant. It may be related to the differences in the measurement method and data processing of HOMA-IR compared to those in other studies. Comparison adjusted funnel plot for the ten outcomes is shown in Figure 6. The comparison adjusted funnel chart for the LSM outcome indicates poor symmetry, suggesting potential publication bias. The reason may be related to the small number of included studies and small sample size. In contrast, the comparison adjusted funnel chart for other nine outcome indicators ALT, CAP, AST, HDL-C, LDL-C, TG, TC, HOMA-IR and BMI showed symmetric distribution in the upper middle part and clustering towards the middle line.

## 8 Discussion

With the changes in human lifestyle and the rapid development of society, the incidence of metabolic syndrome, including obesity and diabetes, is increasing annually. NAFLD, as a hepatic manifestation of metabolic syndrome, has become one of the leading causes of chronic liver disease (21). Furthermore, NAFLD is also a risk factor for cardiovascular disease, and atherosclerotic cardiovascular disease is also a leading cause of death in NAFLD patients (22, 23). Despite the dangers of NAFLD, few drugs are used to treat it. Healthy lifestyle and weight loss remain effective interventions to prevent and improve NAFLD. Evidence indicates that a 10% weight loss can reduce liver damage-levels and improve liver steatosis and fibrosis in NASH (24). Recently, studies have shown the potential mechanism of intestinal microbiota in NAFLD and the benefits of modulating the intestinal microbiome for NAFLD, which indicates the promising effect of MTTs on NAFLD/NASH treatment (25, 26).

This system review summarized the data from 37 randomized controlled trials, comparing synbiotics, probiotics, prebiotics, fecal transplantation, and antibiotics on liver enzymes (ALT, AST), glycolipid metabolism (TG, TC, LDL-C, HDL-C, HOMA-IR), and non-invasive steatosis and fibrosis in the liver (CAP, LSM) in 1921 patients with NAFLD. One study reported the effects of antibiotics on NAFLD, and network meta-analysis was not feasible, so the aim was to evaluate the quality and limitations of this study. The funnel plots showed that data points distributed relatively symmetrical and concentrated in the upper-middle area, indicating the reliablity of the data.

Our study found that probiotics and synbiotics were significantly superior to placebo in reducing liver enzyme markers AST and ALT, consistent with previous findings (27, 28). However, some studies have found that synbiotics cannot reduce ALT, and this may be related to the quantity of included articles (29). In addition, we found that synbiotics were also superior to conventional treatment in reducing AST, where conventional treatment includes diet management, exercise, weight loss, etc. Of the various interventions, there was no particular intervention that was superior to the others (30).

Our study found that probiotics and synbiotics were beneficial in improving liver steatosis and fibrosis. We evaluated the CAP score and liver stiffness measurement, which provide a quantitative, non-invasive evaluation of NAFLD by measuring hepatic steatosis and fibrosis. We found that probiotics and synbiotics could significantly reduce LSM. This may be related to the improvement of inflammatory response. Inflammatory factors such as IL-1*β* and TNF-*α* activate hepatic stellate cells in the liver to induce their differentiation into fibroblast-like cells, resulting in excessive deposition of a large amount of extracellular matrix in the liver, increasing liver stiffness. Previous studies have shown that probiotics can treat inflammatory diseases by regulating the release of intestinal inflammatory factors and increasing the secretion of anti-inflammatory factor IL-10 (31, 32). Furthermore, synbiotic supplementation has shown a better effect in reducing LSM compared to probiotics. Nevertheless, we cannot draw safely conclude that probiotics and synbiotics supplementation can improve fibrosis, considering the association between the reduced ALT levels and LSM (33). Synbiotics supplementation has shown superior function in reducing the CAP indicator, consistent with other meta-analyses (30). Synbiotics is the combination of probiotics and prebiotics in a formulation and, as such, has the advantage of producing increased levels of butyrate, which can upregulate GLP-1R expression to decrease hepatic steatosis (34). In addition, Alves et al. found that synbiotics can alter the expression of genes related to β-oxidation and lipogenesis (16).

Our study also found that probiotics was capable of reducing BMI, consistent with other studies (35). A study has shown that BMI reduction is dependent on NAFLD improvement, indicating that probiotics is a promising treatment for weight loss (36). HOMA-IR is a widely used model method for evaluating insulin resistance. Insulin resistance leads to increased insulin and blood glucose levels, reducing glucose uptake and increasing peripheral tissue decomposition. In this process, fatty acid accumulation in the liver and glucose metabolism disorders develop, contributing to NAFLD (37). We found that probiotics and Allogenic FMT could improve insulin resistance. We included 14 studies to evaluate the effect of MTTs on improving HOMA-IR. The inconsistency test (*p* < 0.05) indicated contradictions and irrationalities among different treatment measures. Subsequently, we employed the node-splitting method to explore the sources of inconsistency and found local inconsistencies in the comparisons between placebo and prebiotics, as well as between probiotics and prebiotics. Finally, using the stepwise exclusion method, we excluded the article by Javadi, after which the inconsistency test indicated no significant difference (*p* > 0.05). Javadi et al. (20) conducted a 12-week placebo-controlled trial comparing the efficacy of prebiotics, probiotics, and synbiotics in treating NAFLD. The measurement formula for HOMA-IR in this study differed from those in other studies, possibly due to the authors’ team using a formula adjusted for a specific human population. Additionally, the limited number of studies incorporating Allogenic FMT might affect the credibility of our conclusion regarding the improvement of HOMA-IR by MTTs.

Compared with other meta-analyses on MTTs, our study evaluated the effect of FMT and antibiotics on NAFLD. Interestingly, we did not find a positive effect of FMT on NAFLD, although there are a few studies indicating that FMT could alleviate high-fat-induced steatohepatitis and improve insulin sensitivity, which were correlated with an increase in the tight junction of small intestinal and butyrate-producing bacteria (12, 13). It may be related to the limitation of quantity of articles, and more RCT studies and long-term follow-ups are needed to verify their efficacy. Only one study evaluated the efficacy of 6-month rifaximin therapy in NAFLD patients. Rifaximin is an oral, non-absorbable antibiotics that reduces endotoxin absorption and intestinal bacterial overgrowth. Abdel-Razik et al. (38), conducted a randomized, double-blind experiment over a 6-month period to observe the effects of rifaximin on NASH. The group receiving the rifaximin for 6 months showed significant improvement (*p < 0.05*) in markers such as ALT, AST, and HOMA-IR compared to the placebo group. However, there were no changes in BMI, cholesterol, or TG. The number of cumulative adverse events between the placebo and rifaximin participants showed no significant difference. Some limitations of this study include the small sample size and the lack of a second liver biopsy to access the liver histopathology changes. Finally, we also focused on the adverse events of antibiotics, which included diarrhea, abdominal pain, nausea, and these may limit their widespread clinical application.

There were a few limitations to our study. First, the length of treatment varied. Most treatment cycles were 12 weeks and 24 weeks, and a few studies had treatment cycles of 8 weeks, which might cause clinical heterogeneity. Regarding the form of probiotics, synbiotics and prebiotics, only four studies explicitly stated that VSL#3 was used as an interventinal strategy, whereas others studies did not clearly specify the types, which may cause bias. Due to the limited number of included studies, we did not conduct a dose subgroup analysis, which may affect the accuracy of the results. Additionally, there were few studies on FMT and antibiotics for the NAFLD treatment, potentially influencing conclusions about their efficacy. Second, included studies were all small sample, reducing the satistical reliability. Third, there was a lack of long-term follow-up data, potentially impacting conclusion. The number of RCTs for FMT (3 RCTs) and antibiotics (1 RCT) was limited, and the results of the NMA merger may not be convincing enough. Given these limitations, it is recommended that future studies should note the following three points:1. It is suggested to carry out multi, large sample studies to clarify the exact effcacy of MTTs in the treatment of NAFLD. 2. It is recommended to conduct long-term follow-up RCT studies to obtain reliable data. 3. It is necessary to clarify the intervention measures to enhance the accuracy of the research conclusions.

In conclusion, we found that synbiotics and probiotics may significantly improve liver function, reduce enzyme levels, and ameliorate hepatic steatosis and fibrosis in patients with NAFLD. Thought-provokingly, sarcopenia, a condition shared by various diseases, was associated with higher risk of developing severe NAFLD (39). Growing evidence highlights the importance of the microbiota in the gut-brain-muscle axis, which is characterized by the involvement of gut flora that regulates skeletal muscle energy and muscle fiber conversion through its metabolites (40). A recent study showed that administration of prebiotics significantly improved muscle function, suggesting prospects for analyzing the MTTs efficacy on NAFLD with sarcopenia (41). And we found synbiotics provided the best effect on LSM reduction. However, no specific evidence was obtained from our study that antibiotics could improve patients with NAFLD due to the limited number of RCT. Finally, common adverse events such as diarrhea, abdominal pain, nausea, etc. should be noted, as they may limit the widespread application of MTTs.

## Data Availability

The original contributions presented in the study are included in the article/Supplementary material, further inquiries can be directed to the corresponding authors.

## Footnotes

^1^ https://www.crd.york.ac.uk/prospero/
