# Supplementary material for: The effect of gut microbiome-targeted therapies in nonalcoholic fatty liver disease: a systematic review and network meta-analysis
Source: Front Nutr. 2025 Jan 6;11:1470185. doi: 10.3389/fnut.2024.1470185 (PMC11743284; doi:10.3389/fnut.2024.1470185)

Supplementary Material

Supplementary Figures

**Supplementary Table 1.** The figure legends are required to have the same font as the main text, 12 point normal Times New Roman, single spaced. Please use a single paragraph for each legend and prepare the figures keeping in mind the PDF layout.

| Database | NO. | Search algorithm | Item found |
| --- | --- | --- | --- |
| pubmed | #1 | "Non-alcoholic Fatty Liver Disease"[Mesh] | 24904 |
|  | #2 | Nonalcoholic[Title/Abstract]) OR (Liver, Nonalcoholic Fatty[Title/Abstract]) OR (Livers, Nonalcoholic Fatty[Title/Abstract]) OR (Nonalcoholic Fatty Liver[Title/Abstract]) OR (Nonalcoholic Fatty Livers[Title/Abstract]) OR (Nonalcoholic Steatohepatitis[Title/Abstract]) OR (Nonalcoholic Steatohepatitides[Title/Abstract]) OR (Steatohepatitides, Nonalcoholic[Title/Abstract]) OR (Steatohepatitis, Nonalcoholic[Title/Abstract]) | 43635 |
|  | #3 | #1 OR #2 | 47007 |
|  | #4 | "Fecal Microbiota Transplantation"[Mesh] | 2896 |
|  | #5 | (Fecal Microbiota Transplantations[Title/Abstract]) OR (Microbiota Transplantation, Fecal[Title/Abstract]) OR (Microbiota Transplantations, Fecal[Title/Abstract]) OR (Transplantation, Fecal Microbiota[Title/Abstract]) OR (Transplantations, Fecal Microbiota[Title/Abstract]) OR (Fecal Microbiota Transplant[Title/Abstract]) OR (Fecal Microbiota Transplants[Title/Abstract]) OR (Microbiota Transplant, Fecal[Title/Abstract]) OR (Microbiota Transplants, Fecal[Title/Abstract]) OR (Transplant, Fecal Microbiota[Title/Abstract]) OR (Transplants, Fecal Microbiota[Title/Abstract]) OR (Fecal Microbiome Transplantation[Title/Abstract]) OR (Fecal Microbiome Transplantations[Title/Abstract]) OR (Microbiome Transplantation, Fecal[Title/Abstract]) OR (Microbiome Transplantations, Fecal[Title/Abstract]) OR (Transplantation, Fecal Microbiome[Title/Abstract]) OR (Transplantations, Fecal Microbiome[Title/Abstract]) OR (Fecal Transplant[Title/Abstract]) OR (Fecal Transplants[Title/Abstract]) OR (Transplant, Fecal[Title/Abstract]) OR (Transplants, Fecal[Title/Abstract]) OR (Donor Feces Infusion[Title/Abstract]) OR (Donor Feces Infusions[Title/Abstract]) OR (Feces Infusion, Donor[Title/Abstract]) OR (Feces Infusions, Donor[Title/Abstract]) OR (Infusion, Donor Feces[Title/Abstract]) OR (Infusions, Donor Feces[Title/Abstract]) OR (Intestinal Microbiome Transplant[Title/Abstract]) OR (Intestinal Microbiome Transplants[Title/Abstract]) OR (Microbiome Transplant, Intestinal[Title/Abstract]) OR (Microbiome Transplants, Intestinal[Title/Abstract]) OR (Transplant, Intestinal Microbiome[Title/Abstract]) OR (Transplants, Intestinal Microbiome[Title/Abstract]) OR (Intestinal Microbiota Transfer[Title/Abstract]) OR (Intestinal Microbiota Transfers[Title/Abstract]) OR (Microbiota Transfer, Intestinal[Title/Abstract]) OR (Microbiota Transfers, Intestinal[Title/Abstract]) OR (Transfer, Intestinal Microbiota[Title/Abstract]) OR (Transfers, Intestinal Microbiota[Title/Abstract]) OR (Intestinal Microbiota Transplantation[Title/Abstract]) OR (Intestinal Microbiota Transplantations[Title/Abstract]) OR (Microbiota Transplantation, Intestinal[Title/Abstract]) OR (Microbiota Transplantations, Intestinal[Title/Abstract]) OR (Transplantation, Intestinal Microbiota[Title/Abstract]) OR (Transplantations, Intestinal Microbiota[Title/Abstract]) OR (Intestinal Microbiome Transplantation[Title/Abstract]) OR (Intestinal Microbiome Transplantations[Title/Abstract]) OR (Microbiome Transplantation, Intestinal[Title/Abstract]) OR (Microbiome Transplantations, Intestinal[Title/Abstract]) OR (Transplantation, Intestinal Microbiome[Title/Abstract]) OR (Transplantations, Intestinal Microbiome[Title/Abstract]) OR (Intestinal Microbiota Transplant[Title/Abstract]) OR (Intestinal Microbiota Transplants[Title/Abstract]) OR (Microbiota Transplant, Intestinal[Title/Abstract]) OR (Microbiota Transplants, Intestinal[Title/Abstract]) OR (Transplant, Intestinal Microbiota[Title/Abstract]) OR (Transplants, Intestinal Microbiota[Title/Abstract]) OR (Intestinal Microbiome Transfer[Title/Abstract]) OR (Intestinal Microbiome Transfers[Title/Abstract]) OR (Microbiome Transfer, Intestinal[Title/Abstract]) OR (Microbiome Transfers, Intestinal[Title/Abstract]) OR (Transfer, Intestinal Microbiome[Title/Abstract]) OR (Transfers, Intestinal Microbiome[Title/Abstract]) OR (Fecal Microbiota Transfer[Title/Abstract]) OR (Fecal Microbiota Transfers[Title/Abstract]) OR (Microbiota Transfer, Fecal[Title/Abstract]) OR (Microbiota Transfers, Fecal[Title/Abstract]) OR (Transfer, Fecal Microbiota[Title/Abstract]) OR (Transfers, Fecal Microbiota[Title/Abstract]) OR (Fecal Transplantation[Title/Abstract]) OR (Fecal Transplantations[Title/Abstract]) OR (Transplantation, Fecal[Title/Abstract]) OR (Transplantations, Fecal[Title/Abstract]) | 4843 |
|  | #6 | #4 OR #5 | 6337 |
|  | #7 | "Synbiotics"[Mesh] | 1148 |
|  | #8 | "Synbiotic"[Title/Abstract] OR "Synbiotics"[MeSH Terms] | 1927 |
|  | #9 | "Probiotics"[MeSH Terms] | 25094 |
|  | #10 | "Probiotic"[Title/Abstract] OR "Probiotics"[MeSH Terms] | 36659 |
|  | #11 | "Prebiotics"[MeSH Terms] | 5032 |
|  | #12 | "Prebiotic"[Title/Abstract] OR "Prebiotics"[MeSH Terms] | 11774 |
|  | #13 | "Anti-Bacterial Agents"[MeSH Terms] | 453811 |
|  | #14 | "agents anti bacterial"[Title/Abstract] OR "anti bacterial agents"[Title/Abstract] OR "antibacterial agents"[Title/Abstract] OR "agents antibacterial"[Title/Abstract] OR "antibacterial agent"[Title/Abstract] OR "agent antibacterial"[Title/Abstract] OR "anti bacterial compounds"[Title/Abstract] OR "anti bacterial compounds"[Title/Abstract] OR (("Compound"[All Fields] OR "compound s"[All Fields] OR "Compounds"[All Fields]) AND "Anti-Bacterial"[Title/Abstract]) OR "anti bacterial agent"[Title/Abstract] OR "agent anti bacterial"[Title/Abstract] OR "anti bacterial agent"[Title/Abstract] OR "anti bacterial compound"[Title/Abstract] OR "anti bacterial compound"[Title/Abstract] OR (("Compound"[All Fields] OR "compound s"[All Fields] OR "Compounds"[All Fields]) AND "Anti-Bacterial"[Title/Abstract]) OR "bacteriocidal agents"[Title/Abstract] OR (("Agent"[All Fields] OR "Agents"[All Fields]) AND "Bacteriocidal"[Title/Abstract]) OR "bacteriocidal agent"[Title/Abstract] OR (("Agent"[All Fields] OR "Agents"[All Fields]) AND "Bacteriocidal"[Title/Abstract]) OR "Bacteriocide"[Title/Abstract] OR "Bacteriocides"[Title/Abstract] OR "anti mycobacterial agents"[Title/Abstract] OR (("Agent"[All Fields] OR "Agents"[All Fields]) AND "Anti-Mycobacterial"[Title/Abstract]) OR "anti mycobacterial agents"[Title/Abstract] OR "anti mycobacterial agent"[Title/Abstract] OR "agent anti mycobacterial"[Title/Abstract] OR "anti mycobacterial agent"[Title/Abstract] OR "antimycobacterial agent"[Title/Abstract] OR (("Agent"[All Fields] OR "Agents"[All Fields]) AND "Antimycobacterial"[Title/Abstract]) OR "antimycobacterial agents"[Title/Abstract] OR "agents antimycobacterial"[Title/Abstract] OR "Antibiotics"[Title/Abstract] OR "Antibiotic"[Title/Abstract] | 449410 |
|  | #15 | #13 OR #14 | 694929 |
|  | #16 | "Randomized Controlled Trials as Topic"[Mesh] | 169325 |
|  | #17 | "Randomized Controlled Trial"[Publication Type] | 605736 |
|  | #18 | #15 OR #16 | 766826 |
|  | #19 | #3 AND #6 AND #17 | 6 |
|  | #20 | #3 AND #8 AND #17 | 13 |
|  | #21 | #3 AND #10 AND #17 | 53 |
|  | #22 | #3 AND #12 AND #17 | 26 |
|  | #23 | #3 AND #15 AND #17 | 6 |
|  | #24 | #19 OR #20 OR #21 OR #22 OR #23 | **104** |

| Database | NO. | Search algorithm | Item found |
| --- | --- | --- | --- |
| Embase | #1 | 'nonalcoholic fatty liver'/exp | 71639 |
|  | #2 | 'non alcoholic fatty liver disease':ab,ti OR 'non alcoholic hepato-steatosis':ab,ti OR 'non alcoholic hepatosteatosis':ab,ti OR 'non alcoholic liver steatosis':ab,ti OR 'non alcoholic steatotic hepatopathy':ab,ti OR 'non-alcoholic fatty liver':ab,ti OR 'non-alcoholic fatty liver disease':ab,ti OR 'non-alcoholic fld':ab,ti OR 'non-alcoholic hepatic steatosis':ab,ti OR 'nonalcoholic fatty liver disease':ab,ti OR 'nonalcoholic fld':ab,ti OR 'nonalcoholic hepatic steatosis':ab,ti OR 'nonalcoholic hepatosteatosis':ab,ti OR 'nonalcoholic liver steatosis':ab,ti OR 'nonalcoholic fatty liver':ab,ti | 49537 |
|  | #3 | #1 OR #2 | 77598 |
|  | #4 | 'antiinfective agent'/exp | 4928219 |
|  | #5 | 'antiinfective agent'/exp OR 'antiinfective agent' OR (antiinfective AND ('agent'/exp OR agent)) OR 'anti bacterial agent':ab,ti OR 'anti bacterial agents':ab,ti OR 'anti infective agents':ab,ti OR 'anti infectives, otic':ab,ti OR 'anti-bacterial agents':ab,ti OR 'anti-infective agents':ab,ti OR 'anti-infectives, otic':ab,ti OR antibacterial:ab,ti OR 'antibacterial agent':ab,ti OR 'antibacterial drug':ab,ti OR 'antibacterial soap':ab,ti OR 'antibacterial spectrum':ab,ti OR 'antiinfectives, otic':ab,ti OR antimicrobial:ab,ti OR 'antimicrobial agent':ab,ti OR 'antimicrobial compound':ab,ti OR 'antimicrobial drug':ab,ti OR 'antimicrobial factor':ab,ti OR antiseptic:ab,ti OR 'antiseptic agent':ab,ti OR 'antiseptic cream':ab,ti OR 'antiseptic foam':ab,ti OR 'antiseptic soap':ab,ti OR 'chemotherapeutic agent':ab,ti OR 'chemotherapeutic drug':ab,ti OR chemotherapeutica:ab,ti OR 'microbiological agent':ab,ti OR 'antiinfective agent':ab,ti | 5034119 |
|  | #6 | #4 OR #5 | 5034119 |
|  | #7 | #3 AND #6 | 14532 |
|  | #8 | #3 AND #6 | 14532 |
|  | #9 | 'fecal microbiota transplantation'/exp | 10012 |
|  | #10 | ('fecal microbiota transplantation'/exp OR 'fecal microbiota transplantation' OR (fecal AND ('microbiota'/exp OR microbiota) AND ('transplantation'/exp OR transplantation)) OR (bacteriotherapy:ab,ti AND feces:ab,ti) OR 'faecal bacteriotherapy':ab,ti OR 'faecal enema':ab,ti OR 'faecal infusion':ab,ti OR 'faecal matter transplant':ab,ti OR 'faecal microbial transplant':ab,ti OR 'faecal microbial transplantation':ab,ti OR 'faecal microbiome transplant':ab,ti) AND 'faecal microbiome transplantation':ab,ti OR 'faecal microbiota transplant':ab,ti OR 'faecal microbiota transplantation':ab,ti OR 'faecal transplant':ab,ti OR 'faecal transplantation':ab,ti OR 'fecal bacterial transplant':ab,ti OR 'fecal bacterial transplantation':ab,ti OR 'fecal bacteriotherapy':ab,ti OR 'fecal enema':ab,ti OR 'fecal infusion':ab,ti OR 'fecal instillation':ab,ti OR 'fecal matter transplant':ab,ti OR 'fecal matter transplantation':ab,ti OR 'fecal microbe transplant':ab,ti OR 'fecal microbial transplant':ab,ti OR 'fecal microbial transplantation':ab,ti OR 'fecal microbiome transplant':ab,ti OR 'fecal microbiome transplantation':ab,ti OR 'fecal microbiota transplant':ab,ti OR 'fecal microbiotal transplant':ab,ti OR 'fecal microflora transplantation':ab,ti OR 'fecal transplant':ab,ti OR 'fecal transplantation':ab,ti OR 'feces bacteriotherapy':ab,ti OR 'feces microbe transplantation':ab,ti OR 'feces microbiota transplantation':ab,ti OR 'feces microflora transplant':ab,ti OR 'feces microflora transplantation':ab,ti OR (fmt:ab,ti AND 'fecal microbiota transplantation':ab,ti) OR 'gut microbial transplant':ab,ti OR 'gut microbial transplantation':ab,ti OR 'gut microbiome transplant':ab,ti OR 'gut microbiome transplantation':ab,ti OR 'gut microbiota transplant':ab,ti OR 'gut microbiota transplantation':ab,ti OR 'gut microflora transplantation':ab,ti OR (imt:ab,ti AND 'intestinal microbiota transplantation':ab,ti) OR 'intestinal microbe transplantation':ab,ti OR 'intestinal microbiota transplant':ab,ti OR 'intestinal microbiota transplantation':ab,ti OR 'intestinal microflora transplantation':ab,ti OR 'rectal bacteriotherapy':ab,ti OR 'stool enema':ab,ti OR 'stool infusion':ab,ti OR 'stool instillation':ab,ti OR 'stool microbial transplantation':ab,ti OR 'stool transplant':ab,ti OR 'stool transplantation':ab,ti OR 'fecal microbiota transplantation':ab,ti | 7035 |
|  | #11 | #9 OR #10 | 11284 |
|  | #12 | #3 AND #11 | 416 |
|  | #13 | 'synbiotic agent'/exp | 3107 |
|  | #14 | 'synbiotic agent'/exp OR 'synbiotic agent' OR synbiotic:ab,ti OR synbiotics:ab,ti OR 'synbiotic agent':ab,ti | 3799 |
|  | #15 | #13 OR #14 | 3799 |
|  | #16 | 'probiotic agent'/exp | 54610 |
|  | #17 | 'probiotic agent'/exp OR 'probiotic agent' OR probiotic:ab,ti OR probiotics:ab,ti OR 'probiotic agent':ab,ti | 63753 |
|  | #18 | #16 OR #17 | 63753 |
|  | #19 | 'prebiotic agent'/exp | 13026 |
|  | #20 | 'prebiotic agent'/exp OR 'prebiotic agent' OR prebiotic:ab,ti OR prebiotics:ab,ti OR 'prebiotic agent':ab,ti | 18879 |
|  | #21 | #19 OR #20 | 18879 |
|  | #22 | #3 AND #15 | 190 |
|  | #23 | #3 AND #18 | 1297 |
|  | #24 | #3 AND #21 | 524 |
|  | #25 | 'randomized controlled trial'/exp | 799881 |
|  | #26 | 'randomized controlled trial'/exp OR 'randomized controlled trial' OR 'controlled trial, randomized':ab,ti OR 'randomised controlled study':ab,ti OR 'randomised controlled trial':ab,ti OR 'randomized controlled study':ab,ti OR 'trial, randomized controlled':ab,ti OR 'randomized controlled trial':ab,ti | 1091935 |
|  | #27 | #25 OR #26 | 1091935 |
|  | #28 | #7 OR #12 OR #22 OR #23 OR #24 | 15467 |
|  | #29 | #27 AND #28 | 908 |

| Database | NO. | Search algorithm | Item found |
| --- | --- | --- | --- |
| Cochrane Library | #1 | MeSH descriptor: [Non-alcoholic Fatty Liver Disease] explode all trees | 1661 |
|  | #2 | (Non alcoholic Fatty Liver Disease or NAFLD or Nonalcoholic Fatty Liver Disease or Fatty Liver, Nonalcoholic or Fatty Livers, Nonalcoholic or Liver, Nonalcoholic Fatty or Livers, Nonalcoholic Fatty or Nonalcoholic Fatty Liver or Nonalcoholic Fatty Livers or Nonalcoholic Steatohepatitis or Nonalcoholic Steatohepatitides or Steatohepatitides, Nonalcoholic or Steatohepatitis, Nonalcoholic):ti,ab,kw | 5005 |
|  | #3 | #1 or #2 | 5005 |
|  | #4 | MeSH descriptor: [Fecal Microbiota Transplantation] explode all trees | 172 |
|  | #5 | (Fecal Microbiota Transplantations OR Microbiota Transplantation, Fecal OR Microbiota Transplantations, Fecal OR Transplantation, Fecal Microbiota OR Transplantations, Fecal Microbiota OR Fecal Microbiota Transplant OR Fecal Microbiota Transplants OR Microbiota Transplant, Fecal OR Microbiota Transplants, Fecal OR Transplant, Fecal Microbiota OR Transplants, Fecal Microbiota OR Fecal Microbiome Transplantation OR Fecal Microbiome Transplantations OR Microbiome Transplantation, Fecal OR Microbiome Transplantations, Fecal OR Transplantation, Fecal Microbiome OR Transplantations, Fecal Microbiome OR Fecal Transplant OR Fecal Transplants OR Transplant, Fecal OR Transplants, Fecal OR Donor Feces Infusion OR Donor Feces Infusions OR Feces Infusion, Donor OR Feces Infusions, Donor OR Infusion, Donor Feces OR Infusions, Donor Feces OR Intestinal Microbiome Transplant OR Intestinal Microbiome Transplants OR Microbiome Transplant, Intestinal OR Microbiome Transplants, Intestinal OR Transplant, Intestinal Microbiome OR Transplants, Intestinal Microbiome OR Intestinal Microbiota Transfer OR Intestinal Microbiota Transfers OR Microbiota Transfer, Intestinal OR Microbiota Transfers, Intestinal OR Transfer, Intestinal Microbiota OR Transfers, Intestinal Microbiota OR Intestinal Microbiota Transplantation OR Intestinal Microbiota Transplantations OR Microbiota Transplantation, Intestinal OR Microbiota Transplantations, Intestinal OR Transplantation, Intestinal Microbiota OR Transplantations, Intestinal Microbiota OR Intestinal Microbiome Transplantation OR Intestinal Microbiome Transplantations OR Microbiome Transplantation, Intestinal OR Microbiome Transplantations, Intestinal OR Transplantation, Intestinal Microbiome OR Transplantations, Intestinal Microbiome OR Intestinal Microbiota Transplant OR Intestinal Microbiota Transplants OR Microbiota Transplant, Intestinal OR Microbiota Transplants, Intestinal OR Transplant, Intestinal Microbiota OR Transplants, Intestinal Microbiota OR Intestinal Microbiome Transfer OR Intestinal Microbiome Transfers OR Microbiome Transfer, Intestinal OR Microbiome Transfers, Intestinal OR Transfer, Intestinal Microbiome OR Transfers, Intestinal Microbiome OR Fecal Microbiota Transfer OR Fecal Microbiota Transfers OR Microbiota Transfer, Fecal OR Microbiota Transfers, Fecal OR Transfer, Fecal Microbiota OR Transfers, Fecal Microbiota OR Fecal Transplantation OR Fecal Transplantations OR Transplantation, Fecal OR Transplantations, Fecal):ti,ab,kw | 1163 |
|  | #6 | #4 or #5 | 1163 |
|  | #7 | #6 and #3 | 30 |
|  | #8 | MeSH descriptor: [Anti-Bacterial Agents] explode all trees | 36581 |
|  | #9 | (Agents, Anti-Bacterial or Anti Bacterial Agents or Antibacterial Agents or Agents, Antibacterial or Antibacterial Agent or Agent, Antibacterial or Anti-Bacterial Compounds or Anti Bacterial Compounds or Compounds, Anti-Bacterial or Anti-Bacterial Agent or Agent, Anti-Bacterial or Anti Bacterial Agent or Anti-Bacterial Compound or Anti Bacterial Compound or Compound, Anti-Bacterial or Bacteriocidal Agents or Agents, Bacteriocidal or Bacteriocidal Agent | 41434 |
|  | #10 | #8 or #9 | 53520 |
|  | #11 | #10 and #3 | 44 |
|  | #12 | MeSH descriptor: [Probiotics] explode all trees | 256 |
|  | #13 | MeSH descriptor: [Synbiotics] explode all trees | 3122 |
|  | #14 | MeSH descriptor: [Prebiotics] explode all trees | 634 |
|  | #15 | #12 or #13 or #14 | 3428 |
|  | #16 | #15 and #3 | 43 |
|  | #17 | #16 or #11 or #7 | 109 |
| Database | NO. | Search algorithm | Item found |
| Web of science | #1 | TS=(Non alcoholic Fatty Liver Disease OR Non alcoholic Fatty Liver Disease OR NAFLD OR Nonalcoholic Fatty Liver Disease OR Nonalcoholic Fatty Liver OR Nonalcoholic Fatty Livers OR Nonalcoholic Steatohepatitis OR Nonalcoholic Steatohepatitides) | 54664 |
|  | #2 | TS=(Agents, Anti-Bacterial OR Anti Bacterial Agents OR Antibacterial Agents OR Agents, Antibacterial OR Antibacterial Agent OR Agent, Antibacterial OR Anti-Bacterial Compounds OR Anti Bacterial Compounds OR Compounds, Anti-Bacterial OR Anti-Bacterial Agent OR Agent, Anti-Bacterial OR Anti Bacterial Agent OR Anti-Bacterial Compound OR Anti Bacterial Compound OR Compound, Anti-Bacterial OR Bacteriocidal Agents OR Agents, Bacteriocidal OR Bacteriocidal Agent OR Agent, Bacteriocidal OR Bacteriocide OR Bacteriocides OR Anti-Mycobacterial Agents OR Agents, Anti-Mycobacterial OR Anti Mycobacterial Agents OR Anti-Mycobacterial Agent OR Agent, Anti-Mycobacterial OR Anti Mycobacterial Agent OR Antimycobacterial Agent OR Agent, Antimycobacterial OR Antimycobacterial Agents OR Agents, Antimycobacterial OR Antibiotics OR Antibiotic ) | 525682 |
|  | #3 | TS=(Fecal Microbiota Transplantations OR Microbiota Transplantation, Fecal OR Microbiota Transplantations, Fecal OR Transplantation, Fecal Microbiota OR Transplantations, Fecal Microbiota OR Fecal Microbiota Transplant OR Fecal Microbiota Transplants OR Microbiota Transplant, Fecal OR Microbiota Transplants, Fecal OR Transplant, Fecal Microbiota OR Transplants, Fecal Microbiota OR Fecal Microbiome Transplantation OR Fecal Microbiome Transplantations OR Microbiome Transplantation, Fecal OR Microbiome Transplantations, Fecal OR Transplantation, Fecal Microbiome OR Transplantations, Fecal Microbiome OR Fecal Transplant OR Fecal Transplants OR Transplant, Fecal OR Transplants, Fecal OR Donor Feces Infusion OR Donor Feces Infusions OR Feces Infusion, Donor OR Feces Infusions, Donor OR Infusion, Donor Feces OR Infusions, Donor Feces OR Intestinal Microbiome Transplant OR Intestinal Microbiome Transplants OR Microbiome Transplant, Intestinal OR Microbiome Transplants, Intestinal OR Transplant, Intestinal Microbiome OR Transplants, Intestinal Microbiome OR Intestinal Microbiota Transfer OR Intestinal Microbiota Transfers OR Microbiota Transfer, Intestinal OR Microbiota Transfers, Intestinal OR Transfer, Intestinal Microbiota OR Transfers, Intestinal Microbiota OR Intestinal Microbiota Transplantation OR Intestinal Microbiota Transplantations OR Microbiota Transplantation, Intestinal OR Microbiota Transplantations, Intestinal OR Transplantation, Intestinal Microbiota OR Transplantations, Intestinal Microbiota OR Intestinal Microbiome Transplantation OR Intestinal Microbiome Transplantations OR Microbiome Transplantation, Intestinal OR Microbiome Transplantations, Intestinal OR Transplantation, Intestinal Microbiome OR Transplantations, Intestinal Microbiome OR Intestinal Microbiota Transplant OR Intestinal Microbiota Transplants OR Microbiota Transplant, Intestinal OR Microbiota Transplants, Intestinal OR Transplant, Intestinal Microbiota OR Transplants, Intestinal Microbiota OR Intestinal Microbiome Transfer OR Intestinal Microbiome Transfers OR Microbiome Transfer, Intestinal OR Microbiome Transfers, Intestinal OR Transfer, Intestinal Microbiome OR Transfers, Intestinal Microbiome OR Fecal Microbiota Transfer OR Fecal Microbiota Transfers OR Microbiota Transfer, Fecal OR Microbiota Transfers, Fecal OR Transfer, Fecal Microbiota OR Transfers, Fecal Microbiota OR Fecal Transplantation OR Fecal Transplantations OR Transplantation, Fecal OR Transplantations, Fecal ) | 10018 |
|  | #4 | TS=(synbiotic OR synbiotics OR synbiotic agent) | 4042 |
|  | #5 | TS=(probiotic OR probiotics OR probiotic agent) | 62757 |
|  | #6 | TS=(prebiotic OR prebiotics OR prebiotic agent) | 23460 |
|  | #7 | #1 AND #2 | 255 |
|  | #8 | #1 AND #3 | 227 |
|  | #9 | #1 AND #4 | 160 |
|  | #10 | #1 AND #5 | 678 |
|  | #11 | #1 AND #6 | 280 |
|  | #12 | #7 AND #8 AND #9 AND #10 AND #11 | 1600 |

**Supplementary Table 2**

| Group | Adverse  Reactions  (1=Yes；0=No） | The number of occurrences | Incidence rate | *P* value |
| --- | --- | --- | --- | --- |
| Probiotics | 1 | 5 | 5/49 | 0.857 |
| Probiotics | 0 | 44 | 44/49 |  |
| Placebo | 1 | 5 | 5/44 |  |
| Placebo | 0 | 39 | 39/44 |  |
| Synbiotics | 1 | 2 | 2/60 | 0.62 |
| Synbiotics | 0 | 58 | 58/60 |  |
| Placebo | 1 | 3 | 3/58 |  |
| Placebo | 0 | 55 | 55/58 |  |

**Supplementary Table 3**

| Indicator | Chi^2^ | *P* |
| --- | --- | --- |
| TG | 4.96 | 0.5485 |
| TC | 2.36 | 0.8842 |
| LDL-C | 5.07 | 0.4073 |
| HDL-C | 8.86 | 0.1146 |
| AST | 4.92 | 0.4257 |
| ALT | 1.86 | 0.7611 |
| BMI | 5.58 | 0.233 |
| HOMA-IR | 17.43 | 0.0006 |

**Supplementary Figure 1**


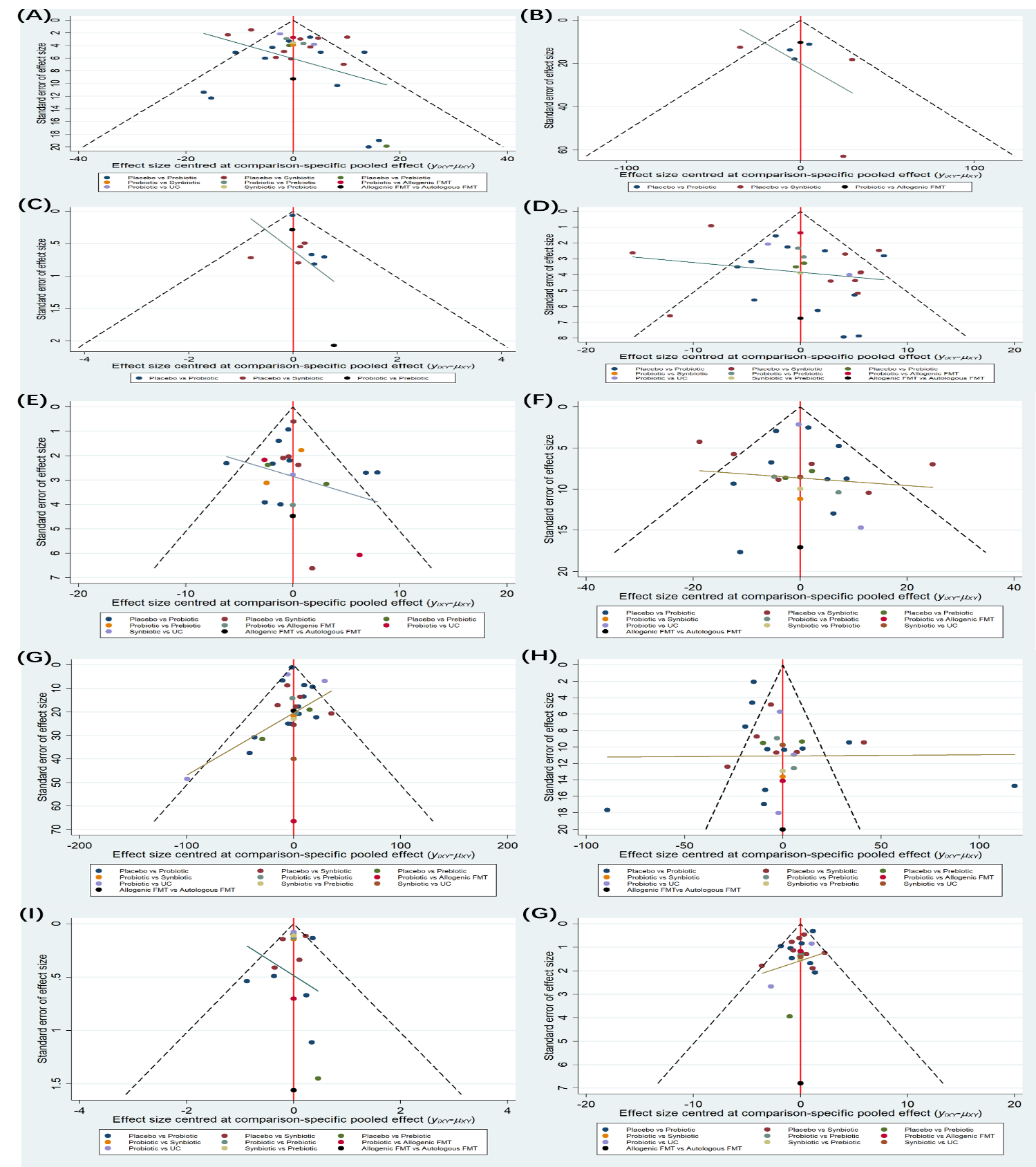

Supplement: Supplementary file 1 [file Table_1.docx]
